# Subjective Symptoms Linked to Sleep Duration: An Analysis from Japanese National Statistics

**DOI:** 10.3390/medicines10110060

**Published:** 2023-11-10

**Authors:** Chikage Kato, Akira Komatsuzaki, Sachie Ono, Asami Iguchi, Kiyoka Arashi, Shiho Motoi, Mio Susuga

**Affiliations:** 1 Department of Dental Hygiene, College at Niigata, The Nippon Dental University, 1-8 Hamaura cho, Chuo-ku, Niigata 951-8580, Japan; chikage@ngt.ndu.ac.jp (C.K.); kiyoka@ngt.ndu.ac.jp (K.A.); hsjc@ngt.ndu.ac.jp (S.M.); mio@ngt.ndu.ac.jp (M.S.); 2 Department of Preventive and Community Dentistry, School of Life Dentistry at Niigata, The Nippon Dental University, 1-8 Hamaura cho, Chuo-ku, Niigata 951-8580, Japan; sachie@ngt.ndu.ac.jp; 3 Department of Dental Anesthesiology, School of Life Dentistry at Niigata, The Nippon Dental University, 1-8 Hamaura cho, Chuo-ku, Niigata 951-8580, Japan; asami@ngt.ndu.ac.jp

**Keywords:** sleep duration, subjective symptoms, disease, sleep quality, quality of life

## Abstract

Background: There is a high prevalence of sleep disorders in Japan, and they are a factor in a decreased quality of life. The main objective of this study was to clarify the background factors of sleep disorders that affect sleep duration, such as subjective symptoms and working hours. Methods: We performed a cross-sectional study on the Japanese national statistics data. Answers from a household questionnaire were used to analyze risk factors for decreases in sleep duration. The subjects were a total of 3972 men and women aged 40–59 years, the age group that forms the core of the working population. For the analysis, a univariate analysis (contingency table) between sleep duration (two groups: sleep duration ≥ 6 h and <6 h) and 42 subjective symptoms was carried out. A multivariate analysis (binomial logistic regression) was conducted using sleep duration and subjective health assessment as objective variables, and odds ratios (ORs) adjusted for sex, working hours, and other factors were obtained. Results: The univariate analysis by subjective symptom showed significant ORs for eight symptoms, including poor sleep quality (OR: 2.24), constipation (OR: 2.24), and dizziness (OR: 1.77). In the multivariate analysis, the model with sleep duration as the objective variable showed significantly adjusted ORs for four variables, including constipation (1.72) and poor sleep quality (1.66). The model with subjective health assessment as the objective variable showed significantly adjusted ORs for eight variables, including dizziness (4.18), while poor sleep quality (1.45) was not significant. Conclusions: The present results suggest the presence of subjective symptoms that may be inferred to be related to decreases in sleep duration.

## 1. Introduction

Sleep disorders such as the inability to ensure sufficient sleep duration or fall asleep have been recognized as major problems at the international level, and in 2013, the American Academy of Sleep Medicine put forward the International Classification of Sleep Disorders (ICSD) [1]. When the World Health Organization (WHO) updated its International Classification of Diseases to the 11th revision (ICD-11), a separate chapter for sleep–wake disorders were included [2], indicating that sleep disorders have now come to be treated as diseases.

However, as far back as the 1940s, there were misgivings that a lack of sleep or disordered sleep habits greatly impacted health, and insufficient sleep duration was recognized as a risk factor for health disorders and disease [3]. Japan has lagged behind in sleep research, and an international comparison by the Organisation for Economic Co-operation and Development (OECD) has ranked Japan among the countries with the shortest sleep duration [4]. Particularly since the 1970s, the decrease in mean sleep duration in Japan [5] has been viewed as a problem, and a large-scale study to investigate the relationship between sleep duration and mortality commenced in 1988 [6].

There was increased interest in the effects of sleep disorders at an international level, with the development of therapies to address sleep apnea proactively in the 1980s [7]. In Japan, a report in 2004 indicated an increased risk of mortality as a result of insufficient sleep [8], and a survey of sleep among working people found that some 2.5–15% of the population felt sleepy during the day and that 23% were aware of their own lack of sleep [9]. From the point of view of the prevention of work-related injury or death, there has been criticism of the attitude that accepts short sleep duration as a matter of individual lifestyle. Japan’s labor management standards aim to improve sleeping hours and rest to a level that is not seen as a problem internationally [10]. 

In light of this situation, the “Health Japan 21” general health promotion strategy set a specific target relating to sleep, referred to as a “Reduction in the percentage of individuals who do not take rest through sufficient sleep” [11]. The Comprehensive Survey of Living Conditions, which provides basic national statistics on health, has included questions relating to sleep since FY2013, allowing comparisons of sleep with factors expected to be related to sleep habits, such as social attributes, lifestyle habits, and employment status. With the permission of the Ministry of Health, Labour and Welfare of Japan to use these anonymized data, the present authors have shown how lifestyle habits such as smoking and alcohol consumption relate to subjective symptoms and regular hospital visits [12,13,14]. It is also important to elucidate the effects of sleep deprivation on lifestyle and QOL.

However, although obtaining sufficient rest through sleep is a major factor in the maintenance of individual health, it has also been pointed out that a lack of sleep and sleep disorders are responsible for significant harm to society in general [15]. In 1993, the US government released a report entitled “Wake Up America: A National Sleep Alert”, in which it put the economic losses arising from sleep disorders and sleepiness at USD 15.9 billion [16]. The problem of sleep disorders thus needs to be addressed urgently, not only at the individual level but also at the societal level.

Therefore, in the present study, we performed an analysis using the most recent national statistics data from middle-aged subjects, who are most at risk from the effects of sleep disorders, to clarify the effects of factors such as subjective symptoms relating to sleep duration and QOL.

## 2. Materials and Methods 

### 2.1. Study Design and Resource Database

A cross-sectional study design was adopted, and data from a single fiscal year (FY 2016) obtained from a government-provided database were used. 

### 2.2. Selection of Subjects and Classification into Sleep Duration Groups

**Figure 1:**
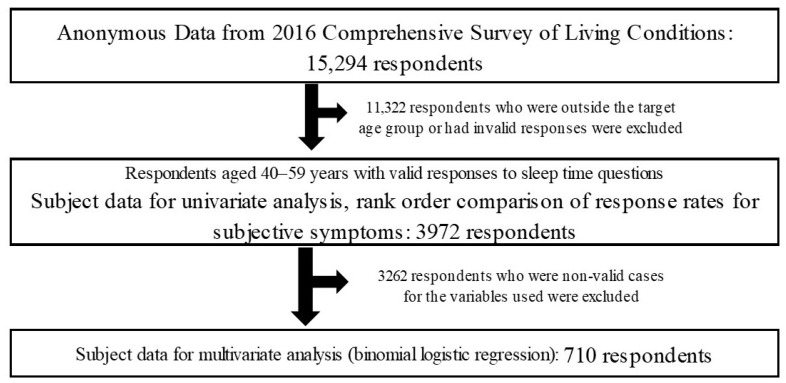
Outline of the data analysis.

The subjects (anonymized data) were 3972 persons (1939 men and 2033 women) from the 40–59 years age group, which is the core of the working population. The subjects were extracted for analysis from the household questionnaire (survey of sex, age, household structure, self-assessed living conditions, etc.) and the health questionnaire (survey of subjective symptoms, outpatient treatments, lifestyle habits, etc.) of the Comprehensive Survey of Living Conditions [12] by the stepwise procedure shown in Figure 1. The subjects for multivariate analysis were 710 respondents who gave valid responses to all the items selected for analysis.

### 2.3. Comparison of Response Rate for Each Survey Item between Sleep Duration Groups

The proportion of the two sleep duration groups responding to each subjective symptom was analyzed using a contingency table (χ^2^ test, univariate odds ratios [ORs], and 95% confidence interval [CIs]) to check for the presence of an association between sleep group and subjective symptoms.

### 2.4. Comparison of the Response Rate Ranking for Each Subjective Symptom between Sleep Duration Groups

The response rate ranking for each symptom was compared between the two sleep groups (sleep duration ≥ 6 h and <6 h) using the Wilcoxon signed-rank test.

### 2.5. Investigation of the Degree of Effect on Sleep Duration and Subjective Health Assessment by Multivariate Analysis (Binomial Logistic Regression)

Binomial logistic regression (complete enumeration method) was performed with subjective symptoms for which an association with sleep duration was found in the contingency table analysis, together with moderator variables such as sex and working hours as explanatory variables and sleep duration (model 1) and subjective health assessment (model 2) as objective variables. Adjusted ORs were obtained for comparison.

### 2.6. Statistical Analysis

Basic data aggregation in this study was carried out using BellCurve (Tokyo, Japan) in Microsoft Office Excel (Redmond, WA, USA) for the χ^2^ tests, the calculation of univariate ORs, the Wilcoxon signed-rank tests, and the binomial logistic regression. The level of significance was set at *p* < 0.05 for all statistical tests.

### 2.7. Ethical Considerations

This study was approved by the Ethical Review Board of the Nippon Dental University College at Niigata (approval No. NDUC-106) and carried out with the permission of the Ministry of Health, Labour and Welfare of Japan (Government Statistics 0413 No. 3) in accordance with Article 36 of the Statistics Act. This study was conducted in accordance with the Declaration of Helsinki, with measures for the protection of personal information in line with the ethical principles for epidemiological studies of the Ministry of Education, Culture, Sports, Science and Technology and the Ministry of Health, Labour and Welfare of Japan. Confirmation of the consent of the survey respondents was carried out by the Ministry of Health, Labour and Welfare (method not disclosed), and data files were provided for use in tabular form following anonymization.

## 3. Results

### 3.1. Classification into Groups by Sleep Duration

There were 1864 subjects (46.9%) with a short sleep duration (sleep duration < 6 h) and 2108 (53.1%) with a longer sleep duration (≥6 h). The following analyses were carried out based on these two groups.

### 3.2. Univariate Analysis of Sex and Other Survey Items with Sleep Duration

Table 1 shows a contingency table of responses, including sex, age, outpatient treatments, working hours, self-assessed living conditions, worries, and stress, effect on everyday healthy lifestyle, and subjective health assessment for each sleep group. Items with high proportions in the <6 h sleep group were subjective health assessment (57.7%) and worries and stress (52.4%); this group also had a higher proportion of women (50.5%) than men (43.2%). Six items were significant in the results of the χ^2^ test and the univariate ORs: sex (univariate OR: 1.34), outpatient treatments (1.17), work time (1.20), self-assessed living conditions (1.15), worries and stress (1.62), and subjective health assessment (1.63). These six items were used as moderating variables in the multivariate analysis.

### 3.3. Univariate Analysis of Responses for Subjective Symptoms and Sleep Duration

Table 2 shows a contingency table analysis of the presence of specific symptoms and the top 20 ranked symptoms (In descending order of response rate) by sleep group.

There were 1216 subjects with some kind of subjective symptom, of whom 640 (52.6%) were in the <6 h sleep group. This was significantly greater than the proportion with no subjective symptoms (*p* < 0.01), with a significant OR (1.39). In the comparison between sleep duration groups of the presence of specific subjective symptoms by means of a χ^2^ test, a significant difference was found for eight symptoms (constipation, not sleeping well, dizziness, irritability, ringing ears, joint pain in the hands/feet, stiff shoulders, and headache), with all showing a significant OR. The symptoms reported with a high proportion in subjects in the short sleep duration group included constipation (70.1%), poor sleep quality (70.0%), and dizziness (65.3%), some of which (constipation and poor sleep quality) showing ORs greater than two and all being statistically significant. In the univariate ORs for other symptoms, only a blocked/runny nose showed an OR less than one, with the OR for all other symptoms greater than one.

One hundred subjects responded that they had the symptom of poor sleep quality, which corresponds to sleep onset disorder or sleep disorder, and this symptom was 15th in the ranking of response rates. Of the subjects who responded that they have poor sleep quality, 70% were in the <6 h group. 

### 3.4. Comparison of Ranking of Response Rates for Subjective Symptoms by Sleep Duration Group

Table 3 shows the ranking of the top 10 response rates for each subjective symptom by sleep duration group. The top four symptoms are the same in both groups, but from the fifth downward, the symptoms differ. The symptom irritability is ranked seventh in the <6 h group.

Although the top four symptoms are ranked the same in both groups, the response rates in the <6 h group were noticeably greater for stiff shoulders (45.5%), lower back pain (39.8%), and headache (20.0%) compared with those in the ≥6 h group.

The Wilcoxon signed-rank test was performed on the ranking of response rates for all 42 symptoms, and a significant difference was found between the two groups.

### 3.5. Multivariate Analyses with Sleep Duration and Subjective Health Assessment as Objective Variables

Given the results of the univariate analysis with the contingency table, multivariate analyses were performed with sleep duration (model one) and subjective health assessment (model two) as objective variables. The adjusted ORs obtained for each model are shown in Table 4.

In the analysis of model one, in which sleep duration was the objective variable, significant adjusted ORs were found for four variables: constipation (1.72), poor sleep quality (1.66), worries and stress (1.39), and stiff shoulders (1.28).

In the analysis of model two, in which subjective health assessment was the objective variable, significant adjusted ORs were found for eight variables, including dizziness (4.18), worries and stress (3.36), and constipation (1.69).

In model two, sleep duration (1.12) and poor sleep quality (1.45) were both included as explanatory variables, but neither showed a significant adjusted OR.

## 4. Discussion

It can be seen from the results of the present study that people with <6 h sleep duration, who have a short sleep duration and a danger of reduced sleep quality, accounted for 47% of the total, and 70% of those who complain of poor sleep quality were included among the people with a short sleep duration.

Sleep is an important activity, accounting for one-third of a person’s lifetime, but perhaps because the qualitative evaluation of sleep is subjective, there are many points that have yet to be clarified [17]. Disordered sleep has long been seen as a problem of lifestyle, but interest in sleep disorders has increased as a result of epidemiological studies finding that not only a huge physical and mental impact [18] but also associations with increased workplace errors and a greater risk of traffic accidents [19]. Ensuring sufficient sleep duration means that a person obtains sufficient rest, and time spent sleeping is important at all life stages. However, during middle age, in particular, which is the core working generation, ensuring sufficient sleep duration is directly connected to how a person works. Those who experience problems with sleep quality may be unable to work satisfactorily, and there is the danger of effects on productivity or work-related accidents. In a 2016 survey of the 26 member OECD countries, Japan was included as one of the countries with the lowest sleep duration [20], and with increasing attention being paid to problems such as death due to overwork in Japan, the relationship between sleep and working patterns has come into greater focus [21]. In Japan, the figurative expression “too busy to sleep” has long been used, but the health risks of chronic sleep deprivation have been identified [22], and the time has surely come to reflect on the work ethic that prioritizes work, even if it means sacrificing sleep duration. The results of this study also show that there were many people whose work was considered to be equivalent to heavy labor from an international perspective.

The present results show that a high proportion of people with <6 h sleep duration have a working week in excess of 56 h, suggesting the need to focus on the relationship between working patterns and sleep duration.

In addition, it has been noted that 27% of the working population of Japan do shiftwork or other types of irregular employment and, as a consequence, face a range of health disorders, including sleep problems. Along with measures to address the mental health of people working long hours, Japan has lagged behind in measures to address short sleep duration [23].

However, the sleep habits of Japanese people have been steadily deteriorating because of the impact of lifestyles, with an increasing trend toward greater irregularity of sleep and nocturnal living [24]. Evidence is being amassed at the international level that sleep disorders increase the risk of disease and worsen prognoses [25], but few epidemiological studies have examined the relationship between sleep disorders and subjective symptoms [26]. Additionally, due to differences in survey methods, comparisons with the results of our study were difficult.

The present results suggest that the presence of subjective symptoms is a factor that aggravates sleep habits, which points to the need for a broad-based descriptive epidemiological analysis. It may be possible to use the results of the Comprehensive Survey of Living Conditions to identify specific symptoms that contribute to the early detection of sleep disorders.

Diseases with a reported tendency to accompany insufficient sleep or sleep disorders include high blood pressure [27], diabetes [28], cardiovascular disease [29], obesity [30], and depression and other mental disorders [31]. In the present study, response rates for fatiguing and accumulative chronic symptoms ranked highest among the subjective symptoms, and thus, there is a concern that the long-term effects of disordered sleep may impact the occurrence of these diseases. Diseases related to sleep include dentistry-related diseases such as sleep apnea [32] and bruxism [33]; however, because the dental symptoms covered in the present study did not include anything relating to these diseases, it has not been possible to analyze them. We also examined the relationship between smoking and drinking habits and sleep duration, but no relationship was detected.

Insufficient sleep duration and sleep disorders are considered to be risk factors for various lifestyle diseases [34], and the physical effects of sleeplessness and fatigue can disrupt the balance of the sympathetic nervous system [35]; therefore, it is likely that insufficient sleep can lead to a range of different symptoms. Past studies comparing sleep and health indices have mainly analyzed functional impairment of the autonomic nervous system and have shown that the effects of a lack of sleep are readily reflected as basic physiological phenomena [36].

It should be noted from the present results that in the multivariate analysis, the adjusted ORs for dizziness and constipation were high and significant, a result that the authors had not anticipated. Dizziness is reported to be intimately related to reflexes and the maintenance of posture [37] and constipation to the physiological rhythm of the gastrointestinal system [38]. These results suggest that they may be hidden factors for sleep disorders.

At the same time, Kageyama et al. [39] analyzed autonomic nervous indices and sleep quality and reported that while sleep quality is related to autonomic nervous indices, no such relationship was found for sleep duration. This indicates the danger of evaluating the effects of sleep disorder on the basis of sleep duration alone.

The present study carried out an analysis on the basis of sleep duration. It was decided to use sleep duration to give uniformity to time indices with working hours, which are the same temporal scale, designed for objectivity in the responses, and operate on the basis of points found in a meta-analysis of different aspects of quality and amount of sleep [40] that uses sleep duration for verification in the field of occupational health. With regard to the feeling of sleep sufficiency, it is by no means universally true to say that sleeping 6 h is sufficient. To assess declines in sleep quality, it appears necessary to have an understanding of numerous background factors, including sleep duration, and we regard the present study as a corroborative report of influences on sleep quality. The analysis results of model two (Subjective health assessment), which examined the relationship between sleep deprivation and QOL, revealed latent risk factors such as constipation.

This study does have some limitations. First, there is the danger of bias because of the reliance on individual subjective evaluations in the questionnaire survey on sleep duration. Second, as this study was an analysis of the results of a cross-sectional survey, it is difficult to verify causal relationships, and the results may have been influenced by unknown confounding factors that were not foreseen. In addition, the relationship between sleep duration and subjective symptoms is likely to be bidirectional, and care must be taken when evaluating cross-sectional studies.

There are limits to the questions that are asked in the national statistics survey, and it is possible that not all the subjects were asked the appropriate survey items. We hope to overcome these issues by carrying out repeated, multifaceted analyses to clarify in a comprehensive fashion how sleep habits relate to bodily and oral symptoms.

In addition, the study did not investigate specific problems associated with different types of sleep disorders [41] or the effects of underlying diseases (including medication) on sleep duration [42]. We hope to continue our investigations from an epidemiological standpoint using other available national statistics data to focus on other factors relating to sleep habits in the future.

## 5. Conclusions

The results of an investigation of sleep duration and subjective symptoms using data from the Comprehensive Survey of Living Conditions showed that sleep duration relates to specific symptoms, as well as to working hours and stress. These results suggest the existence of specific symptoms that may negatively affect sleep duration and point to the importance of emphasizing self-assessment of sleep habits as part of self-monitoring of health in future occupational health measures for lifestyle diseases. 

## Figures and Tables

**Table 1:** Results of the univariate analysis of sex and other survey items with sleep time.

Item	Response (^§^)	<6 h	(%)	≥6 h	(%)	Total	(%)	χ^2^ Test	Odds Ratio	95% CI
Sex	Woman (1)	1027	(50.5)	1006	(49.5)	2033	(100.0)	**	1.34	1.19–1.52
	Man (0)	837	(43.2)	1102	(56.8)	1939	(100.0)			
Age	50s	897	(47.1)	1007	(52.9)	1904	(100.0)		1.01	0.89–1.14
	40s	967	(46.8)	1101	(53.2)	2068	(100.0)			
Outpatient treatment	Yes (1)	721	(49.5)	737	(50.6)	1458	(100.0)	*	1.17	1.03–1.33
	No (0)	1137	(45.5)	1361	(54.5)	2498	(100.0)			
Working hours per week	≥56 h (1)	562	(50.2)	558	(49.8)	1120	(100.0)	**	1.20	1.05–1.38
	<56 h (0)	1274	(45.6)	1522	(54.4)	2796	(100.0)			
household economy	Difficult (1)	1089	(48.4)	1159	(51.6)	2248	(100.0)	*	1.15	1.01–1.30
	Normal, Comfortable (0)	775	(49.0)	949	(55.1)	1724	(100.0)			
Worries and stress	Yes (1)	1121	(52.4)	1017	(51.6)	2138	(100.0)	**	1.62	1.43–1.84
	No (0)	743	(40.5)	1091	(55.1)	1834	(100.0)			
Effect on everyday life	Effect	174	(50.9)	168	(49.1)	342	(100.0)		1.18	0.95–1.02
	No effect	1690	(46.6)	1940	(53.4)	3630	(100.0)			
Subjective health assessment	Bad, Not good (1)	266	(57.7)	195	(42.3)	461	(100.0)	**	1.63	1.34–1.98
	Not bad, Good, Very good (0)	1598	(45.5)	1913	(54.5)	3511	(100.0)			

^§^ Set as explanatory variables for the binomial logistic regression (** *p* < 0.01, * *p* < 0.05).

**Table 2:** Univariate analysis of responses to subjective symptoms and sleep time.

Symptom	Response (^§^)	<6 h	(%)	≥6 h	(%)	Total	(%)	χ^2^ Test	Odds Ratio	95% CI
Presence of subjective symptoms	Yes	640	(52.6)	576	(47.4)	1216	(100.0)	**	1.39	1.21–1.59
	No	1214	(44.4)	1520	(55.6)	2734	(100.0)			
Stiff shoulders	Yes (1)	291	(58.0)	211	(42.0)	502	(100.0)	**	1.44	1.15–1.82
	No (0)	349	(48.9)	365	(51.1)	714	(100.0)			
Lower back pain	Yes (1)	255	(55.2)	207	(44.8)	462	(100.0)		1.18	0.94–1.49
	No (0)	385	(51.1)	369	(48.9)	754	(100.0)			
Headache	Yes (1)	128	(58.7)	90	(41.3)	218	(100.0)	*	1.35	1.00–1.82
	No (0)	512	(51.3)	486	(48.7)	998	(100.0)			
Lethargic	Yes (1)	126	(58.6)	89	(41.4)	215	(100.0)		1.34	0.99–1.81
	No (0)	514	(51.4)	487	(48.6)	1001	(100.0)			
Joint pain in hands/feet	Yes (1)	117	(60.2)	76	(39.4)	193	(100.0)	*	1.47	1.08–2.01
	No (0)	523	(51.1)	500	(48.9)	1023	(100.0)			
Blocked/runny nose	Yes (1)	83	(49.4)	85	(50.6)	168	(100.0)		0.86	0.62–1.19
	No (0)	557	(53.2)	491	(46.8)	1048	(100.0)			
Cough, phlegmatic	Yes (1)	87	(53.1)	77	(46.9)	164	(100.0)		1.01	0.73–1.41
	No (0)	553	(52.6)	499	(47.4)	1052	(100.0)			
Blurred vision	Yes (1)	84	(58.3)	60	(41.7)	144	(100.0)		1.29	0.91–1.85
	No (0)	556	(51.9)	516	(48.1)	1072	(100.0)			
Visual impairment	Yes (1)	85	(59.4)	58	(40.6)	143	(100.0)		1.36	0.95–1.95
	No (0)	555	(51.7)	518	(48.3)	1073	(100.0)			
Numb limbs	Yes (1)	82	(58.2)	59	(41.8)	141	(100.0)		1.28	0.91–1.84
	No (0)	558	(51.9)	517	(48.1)	1075	(100.0)			
Irritable	Yes (1)	86	(64.2)	48	(35.8)	134	(100.0)	**	1.71	1.17–2.48
	No (0)	554	(51.2)	528	(48.8)	1082	(100.0)			
Itchiness (eczema, athlete’s foot, etc.)	Yes (1)	68	(53.5)	59	(46.5)	127	(100.0)		1.04	0.72–1.51
	No (0)	572	(52.5)	517	(47.5)	1089	(100.0)			
Swollen/heavy feet	Yes (1)	67	(59.3)	46	(40.7)	113	(100.0)		1.35	0.91–1.99
	No (0)	573	(51.9)	530	(48.1)	1103	(100.0)			
Ringing ears	Yes (1)	68	(63.6)	39	(36.4)	107	(100.0)	*	1.64	1.09–2.47
	No (0)	572	(51.6)	537	(48.4)	1109	(100.0)			
Poor sleep quality	Yes (1)	70	(70.0)	30	(30.0)	100	(100.0)	**	2.24	1.43–3.48
	No (0)	570	(51.1)	546	(48.9)	1116	(100.0)			
Dizziness	Yes (1)	64	(65.3)	34	(34.7)	98	(100.0)	**	1.77	1.15–2.73
	No (0)	576	(51.5)	542	(48.5)	1118	(100.0)			
Constipation	Yes (1)	68	(70.1)	29	(29.9)	97	(100.0)	**	2.24	1.42–3.51
	No (0)	572	(51.1)	547	(48.9)	1119	(100.0)			
Stomach upset/heartburn	Yes (1)	53	(58.9)	37	(41.1)	90	(100.0)		1.32	0.85–2.03
	No (0)	587	(52.1)	539	(47.9)	1126	(100.0)			
Swollen/bleeding gums	Yes (1)	49	(56.3)	38	(43.7)	87	(100.0)		1.17	0.76–1.82
	No (0)	591	(52.3)	538	(47.7)	1129	(100.0)			
Forgetful	Yes (1)	52	(61.2)	33	(38.8)	85	(100.0)		1.45	0.93–2.29
	No (0)	588	(52.0)	543	(48.0)	1131	(100.0)			

^§^ Set as explanatory variables for the binomial logistic regression. (** *p* < 0.01, * *p* < 0.05). The symptoms with the highest response rate up to the 20th are shown.

**Table 3:** Comparison of ranking of response rates for subjective symptoms by sleep time group.

Ranking of Symptom	<6 h: No. of Responses (%) *	≥6 h: No. of Responses (%) *
1st	Stiff shoulders 291 (45.5)	Stiff shoulders 211 (36.6)
2nd	Lower back pain 255 (39.8)	Lower back pain 207 (35.9)
3rd	Headache 128 (20.0)	Headache 90 (15.6)
4th	Lethargic 126 (19.7)	Lethargic 89 (15.5)
5th	Joint pain in hands/feet 117 (18.3)	Blocked/runny nose 85 (14.8)
6th	Cough, phlegmatic 87 (13.6)	Cough, phlegmatic 77 (13.4)
7th	Irritable 86 (13.4)	Joint pain in hands/feet 76 (13.2)
8th	Visual impairment 85 (13.3)	Blurred vision 60 (10.4)
9th	Blurred vision 84 (13.1)	Numb limbs/Itchiness (eczema, athlete’s foot, etc.) 59 (10.2)
10th	Blocked/runny nose 83 (13.0)	Visual impairment 58 (10.1)
Wilcoxon signed-rank test (Comparison of all 42 symptoms)	*p* < 0.001	

* Percentage of respondents with the symptom with respect to the total number in the group.

**Table 4:** Results of the multivariate analysis (binomial logistic regression analysis) with sleep time and subjective health assessment as objective variables.

Analysis Model	Model 1			Model 2		
Objective Variable	Sleep Time (<6 h: 1, ≥6 h: 0)			Subjective Health Assessment (Bad/Not Good: 1, Not Bad/Good/Very Good: 0)		
	Explanatory Variable	Adjusted Odds Ratio	95% C.I.	Explanatory Variable	Adjusted Odds Ratio	95% C.I.
	Constipation	1.72 *	1.07–2.77	Dizziness	4.18 **	2.55–6.87
	Poor sleep quality	1.66 *	1.03–2.66	Worries and stress	3.36 **	2.19–5.14
	Worries and stress	1.39 *	1.04–1.86	Gender ^¶^	1.74 **	1.30–2.32
	Stiff shoulders	1.28 *	1.00–1.64	Outpatient treatment ^¶^	1.74 **	1.31–2.16
				Constipation	1.69 *	1.03–2.77
				Irritable	1.63 *	1.07–2.49
				Working hours ^¶^	1.62 *	1.07–2.16
				Headache	1.48 *	1.03–2.12
Coefficient of determination	R^2^ (Cox–Snell)	0.05		R^2^ (Cox–Snell)	0.16	

Gender and other variables from Table 1 inserted as moderator variables ^¶^ to obtain adjusted odds ratios. (** *p* < 0.01, * *p* < 0.05). Only significant odds ratios >1 are shown.

## Data Availability

Data are contained within the article.
